# Prevalence and development of hip and knee osteoarthritis according to American College of Rheumatology criteria in the CHECK cohort

**DOI:** 10.1186/s13075-018-1785-7

**Published:** 2019-01-05

**Authors:** Jurgen Damen, Rogier M. van Rijn, Pieter J. Emans, Willem K. H. A. Hilberdink, Janet Wesseling, Edwin H. G. Oei, Sita M. A. Bierma-Zeinstra

**Affiliations:** 1 Department of General Practice, Erasmus MC, University Medical Center, PO Box 2040, 3000 CA Rotterdam, the Netherlands; 2 National Institute of Mental Health and Addiction, Trimbos Institute, Utrecht, the Netherlands; 3 Department of Orthopaedic Surgery, Maastricht University Medical Center, Maastricht, The Netherlands; 4 Allied Health Care Center for Rheumatology and Rehabilitation, Groningen, the Netherlands; 5 Dutch association for Rheumatology, UMC Utrecht, Utrecht, the Netherlands; 6 Department of Radiology & Nuclear Medicine, Erasmus MC, University Medical Center, Rotterdam, The Netherlands; 7 Department of Orthopaedics, Erasmus MC, University Medical Center, Rotterdam, the Netherlands

**Keywords:** Osteoarthritis, Diagnostic, Cohort, Hip, Knee, Diagnostic criteria

## Abstract

**Background:**

We aimed to evaluate the prevalence of hip and knee osteoarthritis (HOA and KOA) according to American College of Rheumatology (ACR) criteria among participants with suspected early symptomatic osteoarthritis (OA) in the CHECK cohort. We also assessed whether participants not fulfilling ACR criteria at baseline develop ACR-defined OA at 2-year and/or 5-year follow up, and which baseline factors are associated with this development.

**Methods:**

The CHECK cohort included 1002 subjects with first presentation of knee and/or hip complaints. The primary outcome was onset of HOA and/or KOA according to the ACR criteria, including the clinical classification criteria and the combined clinical and radiographic classification criteria at 2-year and/or 5-year follow up.

**Results:**

Of the participants with hip complaints, 63% (*n* = 370) were classified as having HOA at baseline according to the ACR criteria. Of those not classified with HOA at baseline, 40% developed HOA according to the clinical or combined clinical/radiographic ACR criteria after 2 and/or 5 years. Up to 92% of participants (*n* = 829) with knee complaints were classified as having KOA at baseline; of those not classified with KOA at baseline, 55% developed KOA according to the clinical ACR criteria or the clinical/radiographic ACR criteria after 2 and/or 5 years. The following factors were associated with development of HOA: morning stiffness (OR 2.39; 95% CI 1.14–4.98), painful internal rotation (OR 2.53; 95% CI 1.23–5.19), hip flexion < 115° (OR 2.33; 95% CI 1.17–4.64) and erythrocyte sedimentation rate (ESR) < 20 mm/h (OR 2.94; 95% CI 1.13–7.61). No variables were associated with development of KOA at 2-year and/or 5-year follow up.

**Conclusions:**

A large proportion of persons with hip complaints not fulfilling the ACR criteria at baseline develop HOA after 2 and/or 5 years of follow up. Almost all persons with knee complaints already fulfill the clinical and/or radiographic ACR criteria for OA, and half of the persons not fulfilling criteria at baseline will do so after 5 years of follow up. Several individual ACR criteria for HOA at baseline were associated with the development of HOA at follow up. This association was not proven for KOA, probably because of the small number of subjects developing KOA in this study.

**Electronic supplementary material:**

The online version of this article (10.1186/s13075-018-1785-7) contains supplementary material, which is available to authorized users.

## Introduction

Osteoarthritis (OA) is associated with joint pain and functional limitation and is a leading cause of disability among older people. OA is considered the most common form of arthritis from which 15–18% of the population suffers [1]. Approximately 22% of the general population suffers from knee pain, and knee and hip pain are even more common in older people [2, 3]. This generally leads to consultation with a physician: e.g., 33% of the population with knee pain in the UK consults a general practitioner (GP) in primary care [4]. One reason for consultation is that patients with knee pain are looking for a definite diagnosis [5]. However, no clear clinical diagnostic primary care tools are available. Diagnosis of OA is often based on radiological evidence and/or on recommendations formulated by OA experts active in secondary care [6].

The diagnosis of OA in patients suffering from knee or hip pain in primary care would become easier if well-defined criteria were used. The American College of Rheumatology (ACR) has developed different criteria for the classification of OA of the knee and hip in order to promote uniformity in reporting OA in epidemiological and intervention studies. These criteria were developed using combinations of clinical, clinical/laboratory, and clinical, laboratory and radiographic criteria [7, 8]. Although these criteria were developed primarily for epidemiological purposes rather than for clinical use, the ACR criteria are commonly used as a diagnostic tool in secondary care. Because the criteria were developed in secondary care with patients with (mostly) rheumatoid arthritis (RA) in the control group, these criteria might primarily distinguish patients with OA from patients with RA. Furthermore, it has been suggested that the criteria are probably mainly diagnostic for late-stage OA [9]. More uniform and early diagnosis of OA would provide a better window of opportunity for interventions and a clear diagnosis could also help to motivate patients for often-difficult lifestyle changes involved with such a diagnosis.

The present study aimed to evaluate the prevalence of ACR criteria in subjects with knee and hip complaints and whether they will develop evident OA according to the ACR criteria for hip and knee OA. This study also aimed to determine predictive factors for the development of knee/hip OA according to the ACR criteria, during 5-year follow up. These predictive factors may help to diagnose OA at an earlier stage in primary care and thereby promote earlier treatment according to established guidelines.

## Patients and methods

### Study design

The CHECK (cohort hip and cohort knee) study is a prospective cohort study of 1002 individuals who first presented with knee and/or hip pain. Details of the protocol are published elsewhere and a summary is presented below [10]. No ethical approval is required for a prognostic cohort without interventions in the Netherlands.

### Study population

Patients that potentially fulfilled the inclusion criteria were invited to join the study when they visited their GP. In addition, participants were recruited through advertisements, articles in local newspapers, and via the website of the Dutch Arthritis Association. Individuals were eligible to participate if they had pain and/or stiffness of the knee and/or hip, were aged between 45 and 65 years, and had not yet consulted their physician for these symptoms, or the first consultation was within the preceding 6 months.

First presenters with pathological, previously diagnosed, conditions that obviously explained the existing symptoms (e.g. other rheumatic disease, isolated tendinitis/bursitis, previous hip or knee joint replacement, congenital dysplasia, osteochondritis dissecans, intra-articular fractures, septic arthritis, Perthes’ disease, ligament or meniscus damage, plica syndrome or Bakers’ cysts (sign of more advanced OA)) were excluded. Other exclusion criteria were co-morbidity that precluded physical evaluation and/or follow up for at least 10 years, malignancy in the last 5 years, and inability to understand the Dutch language [10].

Physicians at the participating centers checked whether referred patients and patients from their outpatient clinic, fulfilled the inclusion criteria. All patients underwent radiographic assessment and a physical examination, and filled out an extensive questionnaire at baseline, and at 2-year and 5-year follow up.

### Outcome measures

OA of the hip/knee was determined using the ACR criteria for hip and knee OA [7, 8]. We determined the clinical classification criteria, and the combined clinical and radiographic classification criteria. The clinical classification of OA of the hip was determined using hip flexion measured during physical examination instead of the erythrocyte sedimentation rate (ESR), as ESR was only available at baseline. Therefore, we followed the alternative proposed by the ACR when the ESR is not available; these alternative criteria were reported to be equally sensitive and specific [8].

For the classification of knee OA, the clinical criteria and the combined clinical and radiographic criteria were determined. The criteria were first defined per joint (i.e. left and right hip/knee) separately. A participant was classified as having hip or knee OA when at least one of the two joints fulfilled one of the ACR criteria at 2-year and/or at 5-year follow up for the hip and the knee separately, e.g. patients fulfilling ACR criteria at 2 years and not at 5 years of follow up would be classified as patients with OA.

### Predictors

The predictors assessed were factors available at consultation with the GP, and consisted of demographic factors (age, gender and body mass index (BMI)), anamnestic factors (site of pain, pain score in the last week and morning stiffness), co-morbidity (lower back pain, previous surgery in the knee or hip, use of analgesics, unilateral or bilateral hip or knee pain), factors from physical examination (pain ont hip/knee flexion, pain and reduced range of motion (ROM) on internal rotation (ROM < 15 vs. > = 15°) and hip flexion (ROM > 115 vs. < =115°), presence of Heberden’s nodules, palpable warmth of the knee, patellofemoral grinding, joint line tenderness, bony enlargement of the knee) and simple diagnostic tests such as Kellgren and Lawrence grade (K&L 0 vs. ≥ 1) on conventional radiography and ESR (< 20 mm/h). An overview of all tested variables (19 in the hip cohort and 21 in the knee cohort) is presented in Additional file 1: Table S1.

### Data analysis

To reduce bias and improve efficiency, we performed multiple imputation of missing values at baseline. We generated 10 imputed datasets using chained equations implemented in the R routine MICE. All analyses were done separately on the 10 imputation sets. A weighted mean outcome (as proposed by Rubin) was calculated [11]. Separate logistic regression models were constructed for participants with hip or knee complaints at baseline, but who were not classified at baseline as having OA according to the ACR classification criteria for hip and knee OA. Predictors used are described in Additional file 1: Table S1. Because of the large number of measured predictors a data reduction method was used. Predictors related to the outcome (*p* < 0.2) were divided into five categories (i.e. demographics, complaints and symptoms, co-morbidities, physical examination, and diagnostic interventions). Per category of participants with knee or hip complaints a multiple logistic or linear regression (enter method) analysis was performed with predictors that were univariately associated with the outcome (*p* < 0.2). All predictors selected in the different categories were again entered into the final logistic or linear regression analysis to build the final model (*p* < 0.05). The results are presented as odds ratios (OR) with 95% confidence intervals (CI). Predictive values and likelihood ratios were calculated [12]. All analyses were performed with the SPSS software package (version 22.0.0.0).

## Results

The baseline characteristics of the study population are presented in Table 1. Of the 1002 participants in the CHECK cohort, 79.0% was female and mean age was 55.9 years. Of the total study population, 58.7% (*n* = 588) had hip complaints, either stiffness or pain, at baseline. Of these, 27.6% (*n* = 162) were classified as having hip OA at baseline according to the ACR clinical criteria, 50.0% (*n* = 295) according to the combined clinical/radiographic criteria for hip OA, and 62.9% (*n* = 370) met either one or the other of these criteria. Knee complaints were reported at baseline by 82.7% of patients (*n* = 829), of whom, 81.3% (*n* = 674) were classified as having knee OA at baseline according to the ACR clinical criteria,73.1% (*n* = 606) according to the combined clinical/radiographic criteria for knee OA, and 91.7% (*n* = 760) met either one or the other of these criteria.

**Table 1:** Baseline characteristics of the CHECK study population at baseline

Characteristics	Participants (*n* = 1002)	Knee complaints^a^ (*n* = 829)	Hip complaints^b^ (*n* = 588)
Women, %	79.0	79.6	80.8
Age in years (SD)	55.9 (5.2)	56.0 (5.1)	55.8 (5.3)
BMI (SD)	26.2 (4.1)	26.4 (4.1)	26.1 (4.1)
WOMAC pain (SD)	25.4 (17.2)	25.6 (17.3)	27.2 (17.1)
WOMAC function	23.5 (17.1)	24 (17.3)	25.3 (17.6)
WOMAC stiffness	33.2 (21.1)	33.8 (21.1)	34.8 (21.2)
NRS (0–10) (SD)	3.6 (2.1)	3.6 (2.1)	3.7 (2.1)
Hip pain, %	58.7	50.1	100.0
Knee pain, %	83.0	100.0	71.1
ACR clinical knee OA, (%)		674 (81.3)	
ACR combined knee OA (%)		606 (73.1)	
Clinical/combined knee OA (%)		760 (91.7)	
ACR clinical hip OA (%)			162 (27.6)
ACR combined hip OA (%)			322 (54.7)
Clinical or combined hip OA (%)			370 (62.9)

*SD* standard deviation, *NRS* numeric rating scale (0–10), *BMI* body mass index, *WOMAC* Western Ontario and McMaster Universities Osteoarthritis Index scores (0–100), *ACR* American College of Rheumatology, *OA* osteoarthritis

^a^Participant with either knee, or knee and hip pain

^b^Participant with either hip, or hip and knee pain

### Predictive factors in participants with hip complaints and development of OA according to the ACR criteria

Of the 198 participants with hip complaints that were not classified as having hip OA at baseline according to the ACR clinical and/or combined criteria and were not lost to follow up, 80 fulfilled the ACR criteria at 2-year and/or 5-year follow up. Based on the 19 potential predictive factors measured at baseline, 8 univariately significant factors were included in the final multivariate logistic regression model. This model identified the following baseline factors: morning stiffness (OR 2.39; 95% CI 1.14–4.98, positive likelihood ratio (LR+) 1.56), painful internal rotation (OR 2.53; 95% CI 1.23–5.19, LR+ 1.71), hip flexion < 115° (OR 2.33 95% CI 1.17–4.64, LR+ 1.47) and ESR < 20 mm/h (OR 2.94; 95% CI 1.13–7.61, LR+ 0.77) (Table 2).

**Table 2:** Multivariate regression analysis for hip OA at 2-years and/or 5-year follow up according to the ACR classification criteria (*n* = 198, 80 cases, a priori risk = 0.40)

Baseline characteristics	Analysis per category OR (95% CI)	Multivariate analysis OR(95% CI)	PPV	NPV	LR+	LR-
Demographics						
Age (<= 50vs. > 50 years)	0.53(0.28–1.01)	0.90(0.41–1.99)	0.36	0.48	0.84	1.6
Complaints and symptoms						
Pain last week, NRS	1.15(0.99–1.33)	1.04(0.87–1.26)	na	na	na	na
Morning stiffness hip (yes = 1/no = 0)	**2.60 (1.14–3.71)**	**2.39 (1.14–4.98)**	**0.51**	**0.66**	**1.56**	**0.75**
Comorbidities and interventions						
Knee pain (yes = 1/no = 0)	0.63 (0.33–1.20)	0.71 (0.32–1.55)	0.37	0.51	0.89	1.42
Painkillers (yes = 1/no = 0)	2.01 (1.12–3.59)	1.60 (0.75–3.41)	0.5	0.67	1.47	0.73
Physical examination						
Painful hip internal rotation (yes = 1/no = 0)	**2.59 (1.43–4.67)**	**2.53 (1.23–5.19)**	**0.53**	**0.69**	**1.71**	**0.66**
Hip flexion ROM (0 = > 115° vs. 1 = <= 115°)	**2.00 (1.12–3.67)**	**2.33 (1.17–4.64)**	**0.5**	**0.67**	**1.47**	**0.73**
Diagnostic tests						
ESR < 20 mm/h	**3.54 (1.30–7.13)**	**2.94 (1.13–7.61)**	**0.46**	**0.77**	**1.22**	**0.4**

Bold values indicate significant values (*p* < 0.05) in the final model

*ACR* American College of Rheumatology, *OR* odds ratio, *PPV* positive predictive value, *NPV* negative predictive value, *NRS* numeric rating scale, *ROM* range of motion, *LR+* positive likelihood ratio, *LR-* negative likelihood ratio, *na* not applicable (continuous variable), ESR erythrocyte sedimentation rate

Combinations of these factors provided even higher likelihood ratios. Individuals with both morning stiffness and painful internal rotation had a LR+ of 4.03 (positive predictive value (PPV) 0.73, negative predictive value (NPV) 0.64, negative likelihood ratio (LR-) 0.83). When individuals presented with morning stiffness, painful internal rotation and hip flexion < 115°, the LR+ was 15 (PPV 0.91, NPV 0.63, LR- 0.88). Addition of ESR < 20 mm/h as a predictor did not enhance the predictive value (LR+ 12,66, LR- 0.89, PPV 0.9, NPV 0.61).

### Predictive factors in participants with knee complaints

A total of 64 participants with knee pain were not classified as having knee OA at baseline according to the ACR clinical and/or combined criteria and were not lost to follow up. Of these, 35 fulfilled the ACR criteria at 2-year and/or 5-year follow up. In this group, 21 potential predictive factors were measured at baseline (Additional file 1: Table S1). Age, morning stiffness, joint line tenderness and ESR < 20 mm/h were included in the final multivariate logistic regression model. In this small sample no variable was statistically significant. Morning stiffness in the knee lasting < 30 min had a positive likelihood ratio (LR+) of 4.97 (PPV 0.86, NPV 0.49, LR- 0.08) (Table 3).

**Table 3:** Multivariate regression analysis for knee OA at 2-year and/or 5-year follow up according to the ACR classification criteria (*n* = 64, 35 cases, a priori risk = 0.55)

Baseline characteristics	Analysis per category OR (95% CI)	Multivariate analysis OR (95% CI)	PPV	NPV	LR+	LR-
Demographics						
Age (<=50 vs. > 50 years)	0.37 (0.13–1.36)	0.42 (0.14–1.06)	0.39	0.37	0.53	1.44
Complaints and symptoms						
Morning stiffness knee (yes = 1/no = 0)	6.79 (0.65–51.23)	6.08 (0.53–69.93)	0.86	0.49	4.97	0.08
Physical examination						
Joint line tenderness	1.05 (0.21–5.14)	1.92 (0.34–10.79)	0.73	0.5	2.2	0.8
Diagnostic tests						
ESR < 20 mm/h	0.94 (0.87–1.02)	0.94 (0.85–1.03)	na	na	na	na

*OR* odds ratio, *PPV* positive predictive value, *NPV* negative predictive value, *NRS* numeric rating scale, *ESR* erythrocyte sedimentation rate, *LR+* positive likelihood ratio, *LR-* negative likelihood ratio, *na* not applicable (continuous variable)

*p* < 0.05

## Discussion

This study demonstrates that the majority of patients presenting for the first time with hip pain fulfill the combined ACR hip OA criteria, both clinical or combined ACR criteria, and that 40% of patients not fulfilling those ACR criteria will develop evident OA according to the clinical or combined ACR criteria for the hip after 5 years. For this last subgroup we identified the following predictive factors: morning stiffness, painful internal rotation, hip flexion < 115° and an ESR < 20 mm/h. Combinations of these signs and symptoms have an even higher predictive value. In first presenters with knee pain, up to 92% do fulfill the clinical or combined ACR criteria, at baseline. For this reason, the number of participants with knee symptoms not fulfilling the ACR criteria was, in fact, too small to assess predictors of OA development. This study is unique in having such a large group of first presenters. We would like to argue that the CHECK cohort represents people in (Dutch) primary care presenting for the first time with hip and knee complaints and suspected of having early OA.

We were surprised by the large percentage of participants fulfilling ACR criteria at baseline in participants with hip complaints, and that this was even more pronounced in participants with knee complaints. In a previous open population-based knee pain cohort that included persons with chronic knee pain, 47% were not diagnosed with OA at baseline [13]. This proportion is larger than our proportion of participants without OA at baseline. This difference could be due to the younger age (mean age 45 years) and lower BMI in that cohort. In that same study, the majority (86%) of persons developed OA during the 12-year follow up [13]. In our study, a smaller proportion of participants with pain in the hip (40%) and knee (55%) were diagnosed with either hip or knee OA according to the ACR criteria during follow up. However, this result could be related to the shorter follow-up period in our study.

The predictive factors we identified to be associated with the development of hip OA are consistent with the previous literature. Morning stiffness and limited internal rotation are known predictors for total hip replacement in primary care [14, 15]. Age and pain levels, however, were not statistically significant in the final model in the current study whereas other studies found these to be predictive [14, 15]. This could be explained by our relatively young cohort with generally quite low pain levels (Western Ontario and McMaster Universities Osteoarthritis Index (WOMAC) pain score 27.2 on a scale of 0–100, numeric rating scale (NRS) 3.7, Table 1) such as can be expected in a cohort with early OA. Limited hip flexion and ESR < 20 mm/h were not identified previously as risk factors for the development of HOA but were statistically significant in our final model. A possible explanation could be that higher ESR was related to inflammatory diseases at baseline that were not evident at the time of inclusion.

In contrast to previous studies we were unable to identify predictors of the knee pain that develops into knee OA [16, 17], even when we performed a separate analysis for the clinical and the combined ACR criteria. Also, in these subgroup analyses, no variables were significantly associated with development of knee OA, except for borderline significant results for morning stiffness. The large percentage of patients with knee pain who fulfill the ACR criteria at baseline is probably the main reason for not finding significant predictive factors based on OR. However the predictive values show that morning stiffness would probably have had good prognostic value if we had had greater statistical power.

As expected, the criteria associated with fulfilling the ACR criteria at follow up, in either the combined or separate analysis for the clinical and the combined ACR criteria, were all sub-items of the ACR criteria. This indicates that pain in combination with one or more of these sub-items of the ACR criteria might be indicative of future OA.

There are currently no clear diagnostic criteria for OA in primary care, e.g. the ACR criteria are widely used in epidemiologic research but not validated in primary care. Most discussions focus on the use of radiographic outcomes [18]. For example, Kellgren and Lawrence (K&L) grade ≥ 2 is accepted as a cutoff for OA in epidemiological studies and (possibly) in secondary care. The cutoff of K&L grade ≥ 1 is useful in epidemiologic studies to predict progression, but its use is not advised in primary care because knee radiography has no additional value in the assessment of individual patients with knee pain [19–22]. However, in the present study we chose to examine not only clinical features but also radiographic features, because of the availability and still frequent use of radiography in primary care. Our study clearly showed that radiographic features do not predict fulfillment of ACR criteria, nor when assessed in subgroups of clinical or combined ACR criteria (data not shown.)

The prevalence, incidence, and predictors of the incidence of OA are clinically important findings, because they implicate that most persons aged 45–65 years of age presenting to a GP with no other hip or knee disease could be diagnosed with clinical OA at that time or are prone to developing clinical OA within the following years. This could help to provide a clear diagnosis, which contributes to early treatment according to the guidelines that are available for both hip and knee, whereas undiagnosed knee and/or hip pain is usually treated according to the best insight of the individual physician [23–25]. For patients diagnosed with OA, first-step treatments (e.g. education, lifestyle advice, and acetaminophen) should be started, due to their beneficial effects in the early stage of the disease process [26].

Our study offers a unique population to study hip and knee pain in first presenters, because the patients included are comparable with patients who would present to a primary care physician and therefore this study helps in addressing the diagnostic challenge of hip and knee pain in primary care. A limitation of our study is that a substantial number of variables were tested in the analysis. Due to the limited number of OA cases identified, we could justify testing only 2–5 variables per analysis per category when building the explorative models. However, clinically relevant variables were used (defined prior to our analyses) that were previously applied in epidemiological/clinical research and no new predictors were introduced. Further, data reduction methods were used by means of restrictions based on *p* values by pre-analyzing the predictors in their categories. Other predictors of OA could remain unexposed due to this lack of power.

## Conclusion

The majority of people presenting with hip pain for the first time fulfill the clinical or combined ACR criteria and 40% of the patients not fulfilling those ACR criteria will develop OA according to the clinical or combined ACR criteria for the hip after 5 years. Predictive factors for the development of HOA are morning stiffness, painful internal rotation, hip flexion < 115° and an ESR < 20 mm/h. In first presenters with knee pain, up to 92% already fulfill the clinical or combined ACR criteria. No predictive characteristics were identified for the development of knee OA in those not fulfilling ACR criteria.

## Recommendations

We would suggest that future studies validate whether patients with hip complaints aged > 45 years with the characteristics of morning stiffness, painful internal rotation, hip flexion < 115° and an ESR < 20 mm/h indeed have early OA. It also needs to be validated whether first presenters with knee complaints aged > 45 years indeed have early KOA.

## Additional file

Additional file 1: **Table S1.** All univarately tested variables in the hip and knee cohort.ᅟ(DOCX 23 kb)
