# Supplementary material for: Prevalence and development of hip and knee osteoarthritis according to American College of Rheumatology criteria in the CHECK cohort
Source: Arthritis Res Ther. 2019 Jan 5;21:4. doi: 10.1186/s13075-018-1785-7 (PMC6321652; doi:10.1186/s13075-018-1785-7)
Supplement: Supplementary file 1 — Table S1. All univarately tested variables in the hip and knee cohort.ᅟ(DOCX 23 kb) [file 13075_2018_1785_MOESM1_ESM.docx]

**Table S1**

| Hip | p-value | OR | CI- | CI+ |
| --- | --- | --- | --- | --- |
| **Demographics** |  |  |  |  |
| Age (<=50 vs. >50 years) | 0.05 | 0.53 | 0.28 | 1.00 |
| Gender (men vs. woman) | 0.44 | 0.71 | 0.30 | 1.70 |
| **Complaints and symptoms** |  |  |  |  |
| Knee (pain yes/no) | 0.16 | 0.63 | 0.33 | 1.20 |
| Pain last week, NRS | 0.06 | 1.15 | 0.99 | 1.33 |
| Morning stiffness hip (yes=1/no=0) | 0.02 | 2.06 | 1.14 | 3.71 |
| Resting pain | 0.22 | 2.24 | 0.61 | 8.26 |
| **Comorbidities and interventions** | |  |  |  |
| Complaints in lower spine | 0.26 | 1.53 | 0.73 | 3.19 |
| BMI (continuous) | 0.96 | 1.00 | 0.93 | 1.08 |
| Surgery knee or hip (yes vs.no) | na |  |  |  |
| Analgesics (yes=1/no=0) | 0.02 | 2.01 | 1.12 | 3.59 |
| Bilateral complaints (yes=0 vs. no=1) | 0.25 | 1.47 | 0.77 | 2.80 |
| **Physical examination** |  |  |  |  |
| Painful hip flexion (yes vs. no) | 0.61 | 1.28 | 0.49 | 3.36 |
| Painful hip internal rotation (yes vs. no) | 0.00 | 2.59 | 1.43 | 4.67 |
| Painful hip external rotation (yes vs. no) | 0.33 | 1.99 | 0.47 | 8.37 |
| Internal rotation ROM (<15 vs >=15 degrees) | 0.80 | 1.36 | 0.12 | 15.28 |
| Hip flexion ROM (>115 vs.<=115 degrees) | 0.02 | 2.00 | 1.12 | 3.57 |
| Heberden’s nodes (yes vs. no) | 0.45 | 0.80 | 0.46 | 1.42 |
| **Diagnostic investigation** |  |  |  |  |
| Kellgren & Lawrence (0 vs 1) | 0.71 | 1.19 | 0.47 | 3.00 |
| ESR <20 vs > 20 mm/h | 0.01 | 3.05 | 1.30 | 7.13 |
| **Knee** |  |  |  |  |
| **Demographics** |  |  |  |  |
| Age (<=50 vs. >50 years) | 0.06 | 0.37 | 0.13 | 1.06 |
| Gender (men vs. woman) | 0.32 | 2.02 | 0.51 | 8.00 |
| **Complaints and symptoms** |  |  |  |  |
| Hip (pain yes/no) | 0.91 | 1.06 | 0.39 | 2.88 |
| Pain last week, NRS | 0.62 | 1.08 | 0.80 | 1.46 |
| Morning stiffness knee | 0.11 | 5.79 | 0.66 | 51.24 |
| Resting pain | na |  |  |  |
| Crepitus while squatting | 0.53 | 1.98 | 0.24 | 16.64 |
| **Comorbidities and interventions** | |  |  |  |
| Complaints in lower spine | 0.54 | 1.74 | 0.30 | 10.27 |
| BMI (continuous) | 0.50 | 1.05 | 0.91 | 1.23 |
| Surgery knee or hip (yes vs. no) | na |  |  |  |
| Analgesics (yes=1/no=0) | 0.30 | 2.17 | 0.51 | 9.27 |
| Bilateral complaints (yes=0 vs. no=1) | 0.27 | 0.54 | 0.18 | 1.61 |
| **Physical examination** |  |  |  |  |
| Painful knee flexion yes vs. no | na |  |  |  |
| Painful knee extension yes vs. No | na |  |  |  |
| Palpable warmth knee | na |  |  |  |
| Patellofemoral grinding | 0.19 | 3.22 | 0.55 | 18.76 |
| Heberden’s nodes yes vs. No | 0.74 | 1.20 | 0.41 | 3.55 |
| Joint line tenderness | 0.12 | 2.75 | 0.77 | 9.86 |
| Bony enlargement knee |  |  |  |  |
| **Diagnostic investigation** |  |  |  |  |
| Kellgren & Lawrence (0=0 / 1=1/2) | 0.95 | 1.05 | 0.21 | 5.14 |
| ESR <20 vs > 20 mm/h | 0.86 | 1.16 | 0.22 | 6.27 |
